# Spectral Entropy Can Predict Changes of Working Memory Performance Reduced by Short-Time Training in the Delayed-Match-to-Sample Task

**DOI:** 10.3389/fnhum.2017.00437

**Published:** 2017-08-31

**Authors:** Yin Tian, Huiling Zhang, Wei Xu, Haiyong Zhang, Li Yang, Shuxing Zheng, Yupan Shi

**Affiliations:** Bio-information College, Chongqing University of Posts and Telecommunications Chongqing, China

**Keywords:** spectral entropy, WM performance, SVR, prediction, classification, BCIs

## Abstract

Spectral entropy, which was generated by applying the Shannon entropy concept to the power distribution of the Fourier-transformed electroencephalograph (EEG), was utilized to measure the uniformity of power spectral density underlying EEG when subjects performed the working memory tasks twice, i.e., before and after training. According to Signed Residual Time (SRT) scores based on response speed and accuracy trade-off, 20 subjects were divided into two groups, namely high-performance and low-performance groups, to undertake working memory (WM) tasks. We found that spectral entropy derived from the retention period of WM on channel FC4 exhibited a high correlation with SRT scores. To this end, spectral entropy was used in support vector machine classifier with linear kernel to differentiate these two groups. Receiver operating characteristics analysis and leave-one out cross-validation (LOOCV) demonstrated that the averaged classification accuracy (CA) was 90.0 and 92.5% for intra-session and inter-session, respectively, indicating that spectral entropy could be used to distinguish these two different WM performance groups successfully. Furthermore, the support vector regression prediction model with radial basis function kernel and the root-mean-square error of prediction revealed that spectral entropy could be utilized to predict SRT scores on individual WM performance. After testing the changes in SRT scores and spectral entropy for each subject by short-time training, we found that 16 in 20 subjects’ SRT scores were clearly promoted after training and 15 in 20 subjects’ SRT scores showed consistent changes with spectral entropy before and after training. The findings revealed that spectral entropy could be a promising indicator to predict individual’s WM changes by training and further provide a novel application about WM for brain–computer interfaces.

## Introduction

Working memory (WM) was originally defined as a cognitive mechanism responsible for the temporal maintain and manipulation of new and stored memory information (Baddeley, 2012). WM was considered a limited-capacity, short-term, information retention system (Ma et al., 2014). The original model of WM proposed by Baddeley included three subcomponents: the central executive, the visuospatial sketch pad and the phonological loop (Baddeley and Hitch, 1994). Later, an additional component, namely the episodic buffer, was added to WM. This component could take information from the other three components and from long-term memory, from which a single episodic representation was created and then temporarily preserved in the buffer (Baddeley, 2000). Individuals exhibited varying abilities in WM. For example, one person was able to memorize more information and manipulate the information more effectively than others (Baddeley, 2003). There is no doubt that if someone is confused, his or her memory ability will decline.

Working memory performance prediction has become an interesting topic which received considerable attention in recent years (Rajji et al., 2015; Johannesen et al., 2016; Tumari and Sudirman, 2016). In previous attempts, numerous approaches were proposed to extract reliable features to predict individual WM performance and distinguish the difficulty levels of cognitive tasks, such as alpha power (Myers et al., 2014), absolute power (Klimesch et al., 2006), and wavelet entropy (Zarjam et al., 2013).

Previous studies related to WM using EEG found that upper alpha event-related desynchronization (ERD) and small power were associated with good performance during actual processing of the task (Klimesch et al., 2006). Autoregressive model (Nai-Jen and Palaniappan, 2004) and wavelet entropy (Zarjam et al., 2013) extracted from EEG signals were used frequently to measure and distinguish the levels of WM task difficulty. Similarly, time-frequency characteristics using wavelet transform were applied to evaluate mental workload in WM with EEG signals. It has been demonstrated that the appearance time and the total power extracted from wavelet analysis were effective features to measure mental workload (Murata, 2005). Combining magnetoencephalography and EEG (MEEG) recordings with source reconstruction techniques showed that synchrony was enhanced with increasing memory loads among the frontoparietal regions during memory retention while the individual WM capacity could be forecasted by phase synchronization in a network among frontoparietal and visual regions (Palva et al., 2010). An event-related functional magnetic resonance imaging (fMRI) study found that better WM performance in a Sternberg-type delayed match WM task could be predicted by greater temporo-parietal junction (TPJ) and default mode network (DMN) deactivation during the encoding period (Anticevic et al., 2010).

In recent years, the rapid development of neuroscience facilitated the improvements of brain–computer interfaces (BCIs) which were information transfer systems transforming brain intention into control commands without involving peripheral neural pathways (Mühl et al., 2014; Putze et al., 2014). Originally, BCIs were designed to provide a new way for patients with impaired motor functions to communicate with others. Generally, motor imagery and evoked visual potentials received a lot of attentions in BCIs (Ferrez and Millán, 2008; George and Lécuyer, 2010). Lately, BCIs have become available to anyone who wants or needs them and have been envisaged to monitor cognitive state, such as attention, fatigue, and emotions (Frey et al., 2013; Mühl et al., 2014, 2015; Chanel and Mühl, 2015). Moreover, the WM-related EEG signal was utilized in BCIs (Putze and Schultz, 2010; Mühl et al., 2014).

To date, the above measures have widely been investigated in the analysis of the WM loads (i.e., various difficulty levels) (Murata, 2005; Mühl et al., 2014; Dimitriadis et al., 2015), but the estimation or prediction of individual WM performance has rarely been involved especially in BCIs. Previous literatures also tried to find ways to improve one’s WM ability which could be reflected by predicting performance in carrying out a wide range of cognitive tasks (Chouhan et al., 2015). To some extent, WM performance might be a symbol of individual’s WM ability when carrying out the memory tasks.

Usually memory has been regarded as a personal constant character. However, recent studies revealed that it could be promoted by adaptive and extended training (Klingberg, 2010). The density of cortical dopamine receptors changed after training through test (Mcnab et al., 2009). Previous findings showed that using WM-related fMRI, the training-induced variations were linked to the increased activity in prefrontal and parietal regions (Olesen et al., 2003). Research on children with attention deficit hyperactivity disorder (ADHD) also suggested that WM impairments might be overcome by training and stimulant medication on WM (Holmes et al., 2010).

In this work, we attempted to use a proper and objective feature of retention period in WM EEG as a biomarker to predict individual’s WM performance. The existing EEG-based studies, to our best knowledge, demonstrated that power spectrum of low frequency resting-state EEG was associated with individual’s WM performance. Changes in brain activity could be reflected by the changes of power spectral density (PSD) during performing cognitive tasks (Weiss, 1992; Pachou et al., 2008).

Recently, different entropy concepts have been applied to describe the order state of sequences (Bruhn et al., 2000, 2001; Xu et al., 2013; Zhang et al., 2015). Among them, Shannon entropy has been shown as an effective measure for the predictability of EEG series in describing anesthetic drug effect (Bruhn et al., 2001; Zhang et al., 2015). However, Shannon entropy was not normalized to the total power of EEG. Consequently, the absolute value of Shannon entropy might vary among individual, which hampered the applications in clinical areas. To overcome this drawback, spectral entropy was developed by using the Shannon entropy to the Fourier-transformed signals (Vanluchene et al., 2004). Therefore, the spectral entropies were regarded as features to distinguish high-performance from low-performance groups in WM with SVM classifier and further predicted subjects’ individual WM performance by short-time training with support vector regression (SVR) prediction model in the current study. We assume that spectral entropy could be applied to predict individual WM performance and provide a novel approach for the study of BCI in the future.

## Materials and Methods

### Ethics Statement

The experiment was approved by the ethical committee of Chongqing University of Posts and Telecommunications. Written informed consent was signed prior to participating in the study. Subjects received a monetary compensation after experiment. None of them had cognitive impairments, mental and neurological disorders.

### Subjects

Twenty healthy and right-handed male subjects (mean age 21 years old) participated in the experiment. Subjects were asked to keep relaxed and to restrain wide movements as much as possible during the whole experiments. Subjects were requested to perform a continuous delayed match task consisted of two sessions at three levels of task difficulty. The two sessions were exactly equal and the only distinction between the two sessions was that subjects carried out the first tasks without training but they performed the second tasks after WM training.

### Stimuli and Design

**FIGURE 1:**
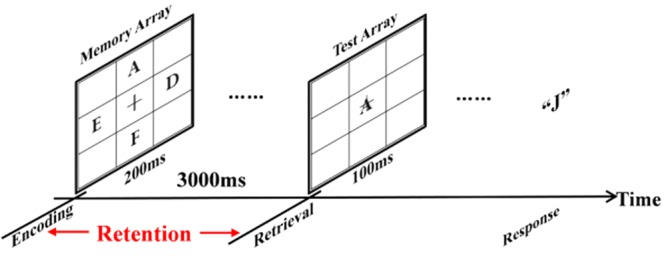
An example of the stimulus sequence in the experiment. Each trial started with a fixation cross (0.5° × 0.5°) at the center of the monitor flashing for 50 ms. Next, the memory array with two, four or eight letters (0.5° × 0.5°) was presented for 200 ms. After 3000 ms retention interval, a probe item was presented for 100 ms and subjects responded with a button press, pointing out if the probe item once was presented in the memory array.

**Figure 1** showed an example of the stimulus sequence. A fixation cross (0.5 × 0.5°; at the center of the monitor) was displayed throughout the entire block of trials. Each trial started with the fixation cross flashing for 50 ms. Following that, the memory array which was randomly consisted of two, four, or eight letters (0.5°× 0.5°) was presented for 200 ms with the same appearing frequency of two, four, and eight letters. After 3000 ms retention interval, the test array was presented on the screen for 100 ms as a probe item. Then subjects responded with a button press, indicating whether the probe item was in the memory array. Subjects pressed the key “F” with their left index fingers for the absence of the probe item from memory array, and pressed the key “J” with their right index fingers for the attendance of the probe item in the memory array. The number between the absence and the attendance of the probe items in the memory array was equal. Response accuracy (RACC) and speed were equally stressed in the instructions.

Subjects were required to maintain central fixation and minimize eye blinks and body motion throughout the recordings. The experiment consisted of two sessions per subject and each session was composed of 60 trials with three kinds of memory loads (two, four, or eight letters). Stimuli were presented and behavioral results were recorded using E-prime software^1^.

### EEG Recordings and Preprocessing

EEG recording was accomplished by using a 64-channel NeuroScan system (Quik-Cap, band pass: 0.05–100 Hz, sampling rate: 1000 Hz, impedances < 5 kΩ) with a vertex reference. To monitor ocular movements and eye blinks, Electro-Oculogram (EOG) signals were simultaneously recorded from four surface electrodes, one pair placed over the higher and lower eyelid and the other pair placed 1 cm lateral to the outer corner of the left and right orbit.

The data was re-referenced to the infinity reference (IR) (Yao, 2001; Tian and Yao, 2013) using the software REST^2^. In the study, EEG was segmented from 100 ms before the onset of the memory array to 100 ms after the subjects’ response onset. In other words, the retention and retrieval stages of WM were extracted for the following preprocessing. EOG and Electromyography (EMG) were excluded by Blind Source Separation (BSS) (Negro et al., 2016). Other noise was removed by automatic artifact rejection (±100 μν). The data was baseline corrected using 100 ms EEG signal before the memory array onset. Then the EEG recordings were filtered with a band-pass of 0.5–45 Hz. After the above preprocessing, 60 trials were obtained for each subject under three memory loads (two, four, and eight items). The retention stage (3000 ms) of each trial was extracted for subsequent analysis.

### Behavioral Analyses

When performing time limit tasks incorporating both reaction time (RT) and RACC, subjects either sacrificed accuracy in exchange for response speed, or exchanged response speed for high accuracy (Mordkoff and Egeth, 1993; Breukelen, 2005). Here, we utilized the Signed Residual Time (SRT) scores (Maris and Han, 2012) for speed accuracy trade-off (Schmitt and Scheirer, 1977) to represent subjects’ behavioral performance. The SRT scoring rule was defined as follows (Schmitt and Scheirer, 1977; Maris and Han, 2012; van Rijn and Ali, 2017):

(1) $$\begin{array}{c} \sum_{i}\left(2{\mathrm{RACC}}_{i}-1\right)(MT-t_{i}) \end{array}$$

where the subscript *i* denoted the item index, RACC*_i_* = {0,1} was the response accuracy (RACC: 0 for incorrect and 1 for correct) for a single trial, and *MT* denoted the maximum allowable response time for item *i*. *t_i_* denoted response latency. The total scores were simply the sum of the scores in each item. In other words, for a correct response, subjects earned the residual time as score, and for an incorrect response, subjects lost the residual time as score.

For testing the left–right hand effect and memory-load effect on behavioral WM performance, 2 (hand: left vs. right) × 3 (memory load: 2 items vs. 4 items vs. 8 items) repeated measures analysis of variances (ANOVAs) were conducted. The dependent variables were the SRT scores in intra-sessions (session 1, session 2) and the change rates of subjects’ SRT scores in inter-sessions (from session 1 to session 2). Greenhouse–Geisser correction was used when the sphericity assumption was violated in repeated measures ANOVAs (Greenhouse and Geisser, 1959), and a factor had more than two levels in the current study to protect against Type I errors (Kisley et al., 2005). In addition, false discovery rate (FDR) was utilized to the correction of multiple comparisons.

### Spectral Entropy

Spectral entropy based on Shannon entropy in physics, quantifying the regularity/randomness of the power spectrum during a given period of time, was used to establish the biomarker for WM performance in the current study. The methodological details were similar to those adopted in a previous study on the prediction of BCI performance (Zhang et al., 2015).

The retention period for each trial was extracted to calculate PSD *P*_sd(f)_ via the Welch’s method (Akbar et al., 2016). The PSD of a time series was defined as the distribution of power as a function of frequency. The normalized PSD was defined as the *P*_sd(f)_ divided by the total power to obtain probability density function.

(2) $$\begin{array}{c} \hat{Psd}\;\;(f)=\frac{Psd\;\;(f)}{\sum_{f\;=\;0.5}^{f\;=\;45}Psd\;\;(f)} \end{array}$$

where _^Psd(f)_ was the normalized PSD of *P*_sd(f)_. We estimated spectral entropy based on the PSD within 0.5–45 Hz. The entropy of _^Psd(f)_ was generated by using the following equation:

(3) $$\begin{array}{c} SEn=-k\sum_{f\;=\;0.5}^{f\;=\;45}\hat{Psd}\;\;(f)\log\;(\hat{Psd}\;\;(f)) \end{array}$$

where *k* = 1. The base of the logarithm was 10 and the unit of *SEn* was dit (i.e., Decimal Digit) in the current study (Schneider, 2007). In effect, spectral entropy reflected the uniformity of the power spectrum distribution. The greater the spectral entropy was, the more uniform the power spectral distribution was.

### Scalp Spatial Distribution for Highest Relationship

**FIGURE 2:**
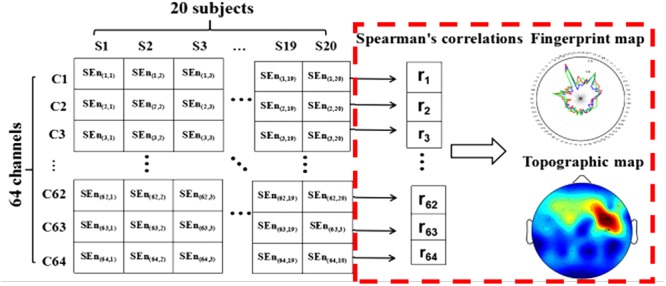
The analysis process of scalp spatial distribution for the relationship between spectral entropy and subjects’ SRT scores.

Spearman’s rank correlation coefficient was widely applied to measure the monotonic relationships. It was defined according to the following equation (Gautheir, 2001; Sedgwick, 2014):

(4) $$\begin{array}{c} r=1-\frac{6\sum_{i=1}^{m}d_{i}^{2}}{m\;\;(m^{2}-1)} \end{array}$$

where each variable was ordered respectively from lowest to highest. *d_i_* was the difference between two ranks for paired variables *x_i_* and *y_i_*. *m* was the number of data pairs. In the current study, the relationships between SRT and spectral entropy on each channobserved. Then, we constructed the fingerprint figures and the scalp spatial distribution according to the spearman’s coefficients on each channel (the processing steps as shown in **Figure 2**). Based on the above method, the electrode site for highest correlation between SRT and spectral entropy was identified to carry out the next steps (see below).

### Grouping Rules and Classification

Subjects were divided into two groups according to their standard z scores of SRT. Here, z-score was defined as:

(5) $$\begin{array}{c} z_{i}=\frac{x_{i}-\mu }{\sigma } \end{array}$$

where *x_i_* (*i* = 1,2,3…,20) was the SRT scores of the *i*th subject, _μ_ was the mean of the 20 subjects’ SRT scores and _σ_ was the standard deviation of SRT scores between 20 subjects in the current study. Subjects whose z values of SRT were above zero were allocated to the group with high WM performance, and the rest were assigned to the group with low WM performance. The high and low memory performance groups were defined as positives and negatives, respectively.

The evaluation of generalization performance for SVM classifier was a required step after using the SVM to classify the high from low memory performance groups. SVM was developed by Vapnik based on statistics learning theory (SLT) (Netherlands, 2008). As a result of its excellent generalization performance, SVM has been applied in a wide variety of issues, such as text classification, images classification, hand writing recognition and gene classification. Furthermore, SVM had the feature of empirical risk minimization (ERM) and global optimum solution (Netherlands, 2008). If the SVM classifier could reflect the relationship between features and the class labels very well, then the classifier was considered that it could predict the classes of new samples with good performance. Therefore, classification accuracy (CA), sensitivity (SE), specificity (SP), and area under ROC curve (AUC) were utilized to evaluate the classification performance of SVM classifier (Galar et al., 2012). At the same time, leave-one-out cross-validation (LOOCV) was applied to evaluate the generalization performance of SVM for a relatively small sample size in the present study.

(1) The percentage of the number of samples predicted correctly in the test set over the total samples, CA, was calculated as follows:

(6) $$\begin{array}{c} CA=\frac{TP-TN}{TP+TN+FP+FN} \end{array}$$

where true positive (*TP*) was the number of high-performance samples correctly predicted and true negative (*TN*) was the number of low-performance samples correctly predicted. False positive (*FP*) denoted the number of high-performance samples incorrectly predicted and false negative (*FN*) denoted the number of low-performance samples incorrectly predicted.

(2) SE and SP were calculated by the following formulae, respectively:

(7) $$\begin{array}{c} SE=\frac{TP}{TP+FN} \end{array}$$

(8) $$\begin{array}{c} SP=\frac{TN}{TN+FP} \end{array}$$

*SE* referred to the ratio of correctly classified high-performance samples to the total population of high-performance samples, whereas *SP* was the ratio of correctly classified low-performance samples to the total population of low-performance samples.

(3) AUC was defined as the area under ROC curves, which was discovered and proved to be better than CA to evaluate the predictive performance of classification learning algorithms (Jin and Ling, 2005). Moreover, AUC was indeed a statistically consistent and more discriminating measure than CA (Ling et al., 2003). Originally, the ROC curves were introduced to evaluate machine learning algorithms (Provost et al., 1997). On ROC curves, *TP* was plotted on the *Y* axis and *FP* was plotted on the *X* axis. It described the classifiers’ performance across the entire range independent of class distributions (Provost et al., 1997; Jin and Ling, 2005). However, often there was no clear dominating relation between two ROC curves in the entire range. Therefore, AUC was introduced to provide a good “summary” for the performance of the learning algorithms based on ROC.

To examine the stability of the SVM classification performance, the classification was performed respectively under intra- and inter-session conditions. In the intra-session, LOOCV was utilized separately for the first and second sessions (Vehtari et al., 2016). One subject was picked out as a test sample, while the rest of the subjects were regarded as the train samples for obtaining the classification model. The above steps were repeated until every subject has been allocated as a test sample for one time. During the inter-session prediction, subjects in the first session were regarded as test samples and subjects in the second session were regarded as the training samples. Then subjects in the first session were considered as training samples and subjects in the second session were considered as test samples.

### Predicting WM Performance

The subject’s spectral entropies derived from WM data at the retention period were selected to predict the corresponding SRT scores. According to the Section “Grouping Rules and Classification,” the spectral entropies of the channel achieving the highest correlations between SRT and spectral entropy were applied to SVR to develop predictive model for a relatively small sample size (Ju et al., 2014) respectively in session 1, session 2, and merged session (i.e., session 1 + session 2). Then, LOOCV was utilized to test the stability of the model and the performance of the predictions. Detailed information for the LOOCV procedure for prediction was described as follows:

We assumed that there were n samples in the dataset. One sample was picked out as a testing set, and the rest of the samples were regarded as training sets to develop the predictive model. The above steps were repeated until every subject had been assigned as a test sample for one time and eventually n SVR models were obtained. The correlation coefficients between predicted and actual SRT scores, together with root-mean-square error of prediction (RMSEP), were calculated to evaluate the prediction performance of the SVR model (Ju et al., 2014; Kichonge et al., 2015).

### Change Rates of SRT Scores and Spectral Entropy between Sessions

For a single subject, behavioral scores usually varied by training. Thus we attempted to study whether the spectral entropy of WM data at retention stage could predict changes in memory performance before and after training.

Since the subjects’ SRT scores and spectral entropy were in different unit scales, a new measure called Change Rate (CR) was defined as follow (Zhang et al., 2015):

(9) $$\begin{array}{c} CR=2\times (TA-TB)/(TA+TB)\times 100\% \end{array}$$

where *TB* denoted the subjects’ SRT scores or spectral entropy of WM data at retention period before training (i.e., session 1). *TA* was the subjects’ SRT scores or spectral entropy predictors at retention period after training (i.e., session 2).

## Results

### Behavioral Performance

For SRT scores, the significant main effects of memory load were separately observed in intra-sessions (session 1: *F* = 218.99, *P* < 0.001, $\eta_{p}^{2}$ = 0.920; session 2: *F* = 162.32, *P* < 0.001, $\eta_{p}^{2}$ = 0.895), indicating that different memory-load tasks affected the behavioral performance on SRT scores regardless of before training and after training. Non-significant main effects of hand were found in intra-sessions (session 1: *F* = 0.435, *P* = 0.518, $\eta_{p}^{2}$ = 0.01; session 2: *F* = 1.51, *P* = 0.234, $\eta_{p}^{2}$ = 0.074), indicating that SRT scores were not affected by left–right hand effect in intra-sessions. There were non-significant interactions between hand and memory load (session 1: *F* = 4.24, *P* = 0.053, $\eta_{p}^{2}$ = 0.183; session 2: *F* = 0.92, *P* = 0.405, $\eta_{p}^{2}$ = 0.046).

For the change rates of SRT scores from session 1 to session 2, there were non-significant main effects of hand (*F* = 3.636, *P* = 0.072, $\eta_{p}^{2}$ = 0.161) and memory loads (*F* = 0.028, *P* = 0.973, $\eta_{p}^{2}$ = 0.002), indicating that the change rates reduced by training were not affected by both hand and memory-load effects. There were non-significant interactions between hand and memory load (*F* = 3.99, *P* = 0.060, $\eta_{p}^{2}$ = 0.169).

### Relationship between SRT Scores and Spectral Entropy

**FIGURE 3:**
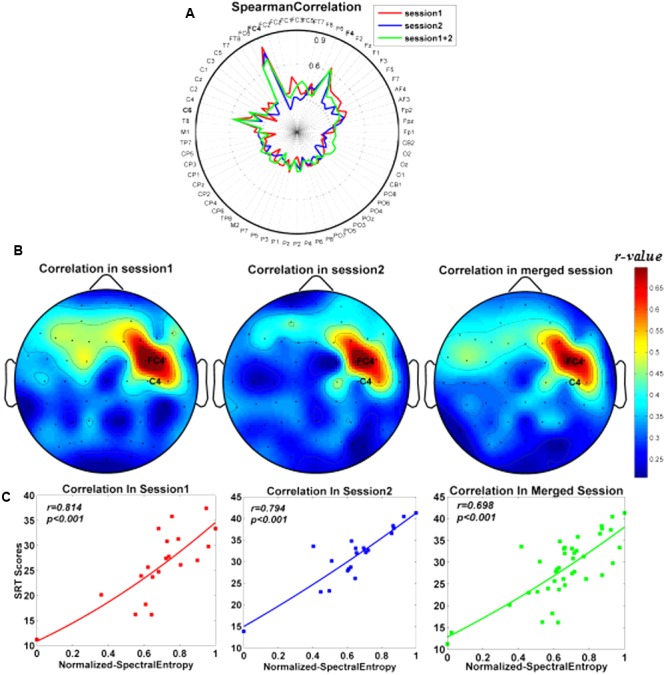
Scalp spatial distribution for correlations between the normalized spectral entropy and subjects’ SRT scores. **(A)** Fingerprint maps. **(B)** Topographic maps. The color bar denoted the correlation values between SRT scores and spectral entropy. **(C)** Correlations between SRT scores and spectral entropy on channel FC4 separately in session 1, session 2, and merged session.

The correlations between SRT scores and the spectral entropy on 64 channels were illustrated in the fingerprint map (**Figure 3A**) and the scalp topographic map (**Figure 3B**). Among them, the spectral entropy on channel FC4 showed the strongest correlation with subjects’ SRT scores (session 1: *r* = 0.814, *P* < 0.001; session 2: *r =* 0.761, *P* < 0.001; and merged session: *r* = 0.698, *P* < 0.001; FDR correction, and also shown in **Figure 3C**).

Therefore, the spectral entropies on channel FC4 from WM EEG recording at the retention stage were then used as features for SVM classifiers to distinguish the high-performance group from the low-performance group, and further used to predict subjects’ SRT scores with SVR prediction model.

### Intra- and Inter-Session Classification for SRT

In the first session (i.e., before training), 11 subjects were divided into high-performance group and nine subjects were divided into low-performance group; while for the second session (i.e., after training), 12 of 20 subjects were assigned to high-performance group and eight subjects were assigned to low-performance group (**Table 1**).

**Table 1:** The number of subjects in different conditions.

Session		Groups		Changes		Change rates	
		High	Low	Increase	Decrease	Consistent	Inconsistent
Intra	Session 1	11	9				
	Session 2	12	8				
Inter				16	4	15	5

*“Groups” denoted the grouping results about the number of subjects separately in session 1 and session 2 according to standard z scores of subjects’ SRT. “Changes” denoted the changes (i.e., SRT scores increased or SRT scores decreased) in the number of subjects after training (i.e., from session 1 to session 2). “Change Rates” denoted the number of subjects whose change rates of SRT showed the consistent and inconsistent change with spectral entropy after training.*

The CA was 95 and 85% for the first and second session with spectral entropy features, respectively. The sensitivity (SE) and specificity (SP) at the optimal operating point, as well as the resulting AUC were showed for the first and second session, respectively. The grouping results were showed in **Table 2**.

**Table 2:** The classification results of SVM classifier.

		CA	AUC	SE	SP
Intra-session	Session 1	0.950	0.976	1.000	0.900
	Session 2	0.850	0.918	0.800	0.900
Inter-session	Session 1^∗^	0.950	0.973	0.898	1.000
	Session 2^∗^	0.900	0.942	0.900	0.900

*CA, classification accuracy; AUC, area under ROC curves; SE, sensitivity; SP, specificity. The session 1 with asterisk (^∗^) denoted the classification results of session 1 with WM data in session 2 as training samples. The session 2 with asterisk (^∗^) denoted the classification results of session 2 with WM data in session 1 as training samples.*

For intra-session prediction, the resulting CA, AUC, SE, and SP were respectively 0.950, 0.976, 1.000, and 0.900 for session 1 with spectral entropy as classification feature. CA, AUC, SE, and SP were respectively 0.850, 0.918, 0.800, and 0.900 for session 2 with spectral entropy as classification feature.

For inter-session classification, CA, AUC, SE, and SP were respectively 0.950, 0.973, 0.898, and 1.000 for session 1 with session 2 as training samples. The resulting CA, AUC, SE, and SP were respectively 0.900, 0.942, 0.900, and 0.900 for session 2 with session 1 as training samples.

**FIGURE 4:**
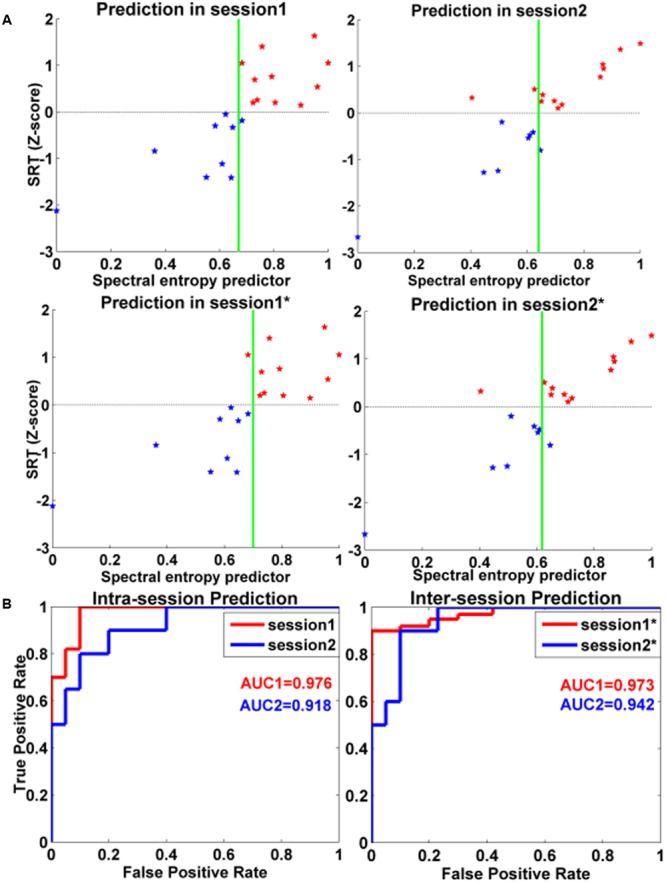
Intra- and inter-session classifications for distinguishing high-performance and low-performance groups. **(A)** Classification plot for intra-session and inter-session. The red star denoted the high-performance group, and the blue star denoted the low-performance group. The green line was the classification boundaries. **(B)** ROC curves for spectral entropy predictors for intra-session and inter-session in classifying the two different performance groups in WM tasks. The session 1 with asterisk (^∗^) denoted the classification results of session 1 with WM data in session 2 as training samples. The session 2 with asterisk (^∗^) denoted the classification results of session 2 with WM data in session 1 as training samples. The abscissa represented the false positive rate, and the ordinate denoted the true positive rate. The red line denoted the ROC curves of session 1, and the blue line represented the ROC curves of session 2. AUC: area under curve.

The classification results were illustrated in **Figure 4** and **Table 2**. The red line demonstrated the ROC curves of session 1, and the blue line showed the ROC of session 2.

### SRT Predicted by the Spectral Entropy

**FIGURE 5:**
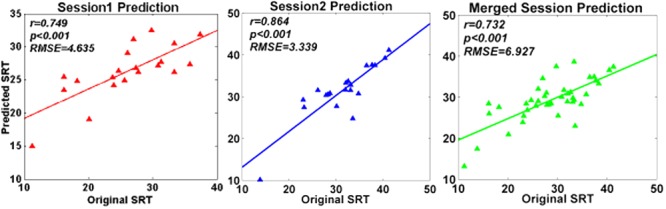
Prediction performance of SVR model. The figures illustrated the correlations between original SRT scores and predicted SRT scores respectively in session 1, session 2, and merged session.

Support vector regression prediction model and LOOCV revealed that the spectral entropy could be utilized to predict individual WM performance on SRT scores in the current study. The RMSEP after doing LOOCV were 4.635 (session 1), 3.339 (session 2), and 6.972 (merged session), respectively. As illustrated in **Figure 5**, the predicting SRT scores were significantly correlated with the original SRT scores (session 1: *r* = 0.749, *P* < 0.001; session 2: *r* = 0.864, *P* < 0.001; merged session: *r* = 0.732, *P* < 0.001; FDR correction).

### Consistent Changes in Spectral Entropy and SRT Scores between Sessions

**FIGURE 6:**
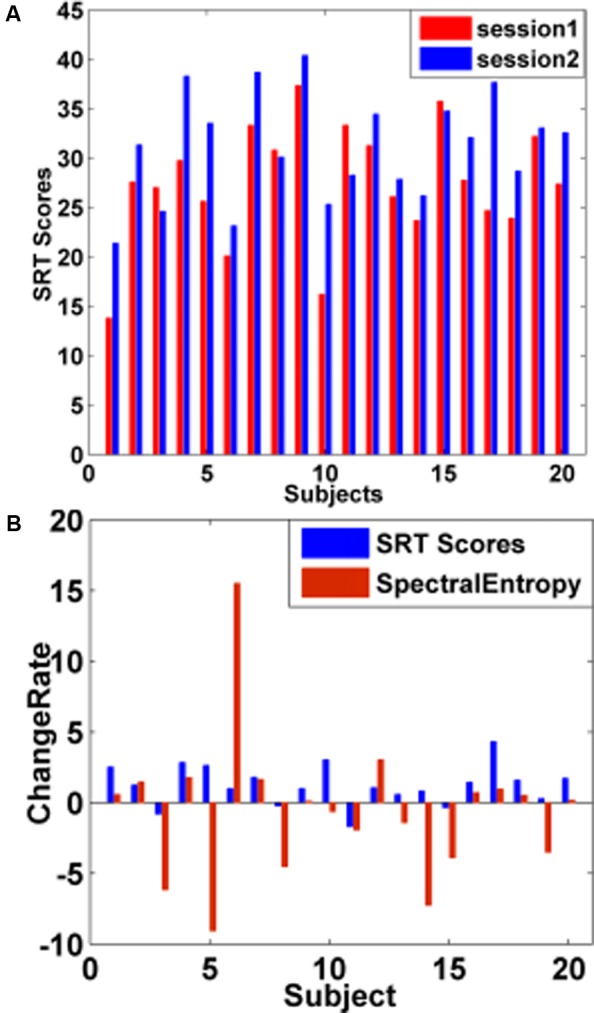
WM performance changes by short-time training. **(A)** Changes in subjects’ SRT scores before and after training. The red bar represented the subjects’ SRT scores before training, and the blue bar denoted the subjects’ SRT scores after training. After training, 16 subjects’ SRT scores increased and 4 subjects’ SRT scores showed a downward trend. **(B)** Change rate (CR) of subjects’ SRT scores and spectral entropies between sessions. The blue bar represented the CR of subjects’ SRT scores, and the red bar denoted the CR of spectral entropies before and after training.

Within single subject, we explored whether the increased (or decreased) changes in spectral entropy could be predictive of the increased (or decreased) changes in SRT scores. **Figure 6A** showed that 16 in 20 subjects’ SRT scores increased by short-time training. **Figure 6B** showed that 15 in 20 subjects’ SRT scores consistently varied with spectral entropy predictor before and after training. The results also could be seen in **Table 1**.

## Discussion

The present study utilized the spectral entropy to predict individual WM performance changes reduced by short-time training during carrying out the delayed-match-to-sample tasks. We found that: (1) the spectral entropy features extracted from FC4 channel during retention stage of WM was strongly related to subjects’ SRT scores; (2) the averaged CA to distinguish high-performance from low-performance group in WM tasks was 90.0 and 92.5% for intra-session and inter-session, respectively; (3) SVR with LOOCV revealed that spectral entropy could predict individual WM performance; (4) 16 out of 20 subjects’ SRT scores increased and 15 in 20 subjects’ SRT scores were consistent with the changes in spectral entropies by short-time training.

### Spatial Distribution for WM Performance

As shown in **Figure 3B**, the spatial distribution of the *r*-values (i.e., the relationship between spectral entropies and behavioral scores) focused on the right frontal area, which was consistent with the previous findings that the frontal cortex might play a prominent part in WM tasks (Gentili et al., 2015; Thürer et al., 2016). The previous study showed that right inferior frontal junction (rIFJ) as a prefrontal cortex (PFC) control region mediated the causal connection between top–down modulation in the service of attentional goals and WM performance (Gazzaley and Nobre, 2012). Among these channels (**Figure 3A**), the spectral entropy of channel FC4 from WM at retention period generated the strongest correlation (*r* = 0.698) in comparison to other channels in the merged session. Likewise, in the two separate sessions, the highest correlation coefficients also appeared on channel FC4 before training (*r* = 0.814) and after training (*r* = 0.794), respectively. The good classification effect of SVM showed that the spectral entropies on channel FC4 from WM EEG recording at the retention stage might be a dependable biomarker to classify two memory groups successfully.

In the current study, there was no significant effect on the change rates of subjects’ SRT scores before and after training when different WM items (i.e., two, four, and eight) were loaded. Thus, subjects’ SRT scores were utilized to reflect individual WM performance while carrying out the WM tasks regardless of memory load. As illustrated by **Figure 3C**, the spectral entropy indexes of the subjects were significantly related to individual SRT scores in all sessions including separate sessions and merged session, indicating that spectral entropy indexes might be applied to predict individual WM performance (i.e., SRT scores).

For the further analysis, SVR prediction model combined with LOOCV was established to estimate the predictive ability of spectral entropy indexes on WM performance separately in session 1, session 2, and the merged session (**Figure 5**). The resultant RMSEP after using LOOCV, as well as the high correlation between original SRT scores and predicted SRT scores, demonstrated that prediction models constructed by SVR were effective. The spectral entropy obtained from channel FC4 could be a biomarker to predict individual WM performance.

### WM Training

Converging evidence revealed that one’s memory ability was absolutely not innate and could be promoted through proper training (Klingberg et al., 2005; Holmes et al., 2010; Klingberg, 2010). Moreover, this improved performance was related to training-induced plasticity from the intracellular level to functional organization of the cortex for WM (Klingberg, 2010). Vogt et al. (2009) conducted a WM training study on multiple sclerosis. They found that the patient’s memory was improved effectively and the spread of bad mood was delayed after receiving the special training for memory (Vogt et al., 2009).

As illustrated by **Figure 6A** and **Table 1**, 16 subjects’ behavioral scores were obviously increased after training, indicating that individuals’ WM performance could be promoted effectively through training, consistent with the previous study (Klingberg et al., 2005; Holmes et al., 2010; Klingberg, 2010). The CR of 15 in 20 subjects’ SRT scores increased (or declined) with the increase (or decline) of the spectral entropy before and after training, which demonstrated that the variations in the spectral entropy predictor could be predictive of the WM performance variations. The findings revealed the consistent changes in SRT scores and spectral entropy by training (**Figure 6B** and **Table 1**).

### WM in BCIs

Previously, P300 in motor imagery tasks and steady state evoked visual potentials (SSVEP) were frequently applied in BCIs (Parra et al., 2003; Dal et al., 2009; George and Lécuyer, 2010). Recently, BCIs also were designed to recognize human emotions (Garcia-Molina et al., 2013; Chanel and Mühl, 2015) and to monitor WM load (Sánchez et al., 2015), while there are still little research on the detection of WM performance or individual memory in BCIs. Consequently, it is of great significance to find a reliable feature for the monitor of individual WM performance in BCIs. The development of predictors on WM performance could recognize the potential memory impairment subjects, assisting them in preparation for the positive life and avoiding the frustration from disordered memory. On the other hand, the relevant study might in turn be heuristic for making effective training strategies for those subjects with low WM performance. Moreover, spectral entropy could be a potential instructive biomarker applied in the detection of schizophrenia, depression, ADHD and two-way affective disorder patients and meanwhile provide a new thought for BCIs on the feature extraction of WM.

### Limitations

As shown in **Figure 6A**, 16 in 20 subjects’ SRT scores obviously was improved after training, while there were still four subjects’ SRT scores representing a downward trend which might be induced by individuals’ state: either their mental state such as easy to feel tense when carrying out the unfamiliar WM tasks or staying unsuitable to experimental environment, or their physical state such as exhaustion without rest well before experiment or easy to be tired when doing the “dull” experiment. For small sample size in the current study, we just utilized LOOCV and four different evaluation indexes of the classifier’s generalization to avoid overfitting as much as possible. It is noted that extracting classification features (like channels) outside of the LOOCV method still could induce overestimation problems. Therefore, we verified the robustness of the selected channel. And the correlation between spectral entropies and WM-related SRT scores of all subjects was highest on channel FC4 regardless of session 1, session 2 and merged session, suggesting the relationship was robust. In addition, spectral entropy was used to predict the individual SRT scores thus indirectly reflecting the WM performance. In the future, a direct way represented the online WM-related BCI would be explored by optimizing classifiers, expanding sample size and improving experimental paradigm.

## Conclusion

In the current study, we first proposed to use spectral entropy as a feature applied in the classification of WM to distinguish high-performance from low-performance groups in the delayed-match-to-sample task. The resulting RMSEP for the SVR prediction models, as well as the high correlations between original SRT scores and predicted SRT scores, demonstrated that the spectral entropies on channel FC4 could implicitly predict individual WM performance. The changes in the spectral entropy can be predictive of changes in behavioral scores for individual WM performance. This study could provide theoretical foundation for researchers in the establishment of enhanced training strategies for memory impairment humans BCIs feedback system on memory state with spectral entropy as feature.

## Author Contributions

Conceived, designed the experiments and wrote the manuscript: YT. Performed the experiments, analyzed the data and wrote the first draft: HuZ. Contributed reagents/materials/analysis tools: WX, HyZ, LY. Discussed the experiment design, analyzed the data and discussed the experiment results: SZ, YS.

## Conflict of Interest Statement

The authors declare that the research was conducted in the absence of any commercial or financial relationships that could be construed as a potential conflict of interest.

## Footnotes

1 http://www.pstnet.com/eprime.cfm

2 www.neuro.uestc.edu.cn/rest
